# The long-lasting legacy of reproduction: lifetime reproductive success shapes expected genetic contributions of humans after 10 generations

**DOI:** 10.1098/rspb.2023.0287

**Published:** 2023-05-10

**Authors:** Euan A. Young, Ellie Chesterton, Virpi Lummaa, Erik Postma, Hannah L. Dugdale

**Affiliations:** ^1^ Groningen Institute for Evolutionary Life Sciences, University of Groningen, Groningen, 9747AG, The Netherlands; ^2^ Faculty of Biological Sciences, School of Biology, University of Leeds, Leeds LS2 9JT, UK; ^3^ Department of Biology, University of Turku, Turku 20014, Finland; ^4^ Centre for Ecology and Conservation, University of Exeter, Penryn TR10 9FE, UK

**Keywords:** fitness proxy, genetic contribution, lifetime reproductive success, human, life history, pedigree

## Abstract

An individual's lifetime reproductive success (LRS) measures its realized genetic contributions to the next generation, but how well does it predict this over longer periods? Here we use human genealogical data to estimate expected individual genetic contributions (IGC) and quantify the degree to which LRS, relative to other fitness proxies, predicts IGC over longer periods. This allows an identification of the life-history stages that are most important in shaping variation in IGC. We use historical genealogical data from two non-isolated local populations in Switzerland to estimate the stabilized IGC for 2230 individuals approximately 10 generations after they were born. We find that LRS explains 30% less variation in IGC than the best predictor of IGC, the number of grandoffspring. However, albeit less precise than the number of grandoffspring, we show that LRS does provide an unbiased prediction of IGC. Furthermore, it predicts IGC better than lifespan, and accounting for offspring survival to adulthood does not improve the explanatory power. Overall, our findings demonstrate the value of human genealogical data to evolutionary biology and suggest that reproduction—more than lifespan or offspring survival—impacts the long-term genetic contributions of historic humans, even in a population with appreciable migration.

## 1. Introduction

Fitness is a fundamental concept in evolutionary biology concerned with how natural selection acts on, for example, genes or phenotypes [1]. To measure how selection acts on either of these units of selection, studies often measure their association with a proxy for fitness. One such proxy is lifetime reproductive success (LRS)—the total number of offspring an individual produces over the course of its lifetime [2]—which captures the realized ability of an individual to contribute genes to the next generation, relative to conspecifics with different genetic or phenotypic variants.

LRS's strength as a proxy for individual fitness stems from (i) not confounding the effects of selection acting on parents and offspring [3–5] and (ii) requiring tracking only one generation of individuals. LRS is therefore one of the most widely used fitness proxies for the estimation of the strength and direction of natural selection in both free-living and captive populations [6]. However, we have little understanding of the extent to which LRS predicts the genetic contributions of individuals beyond initial generations [7] and how this compares to other fitness proxies (but see [8]), or in other words, which part(s) of an individual's life history are the key determinants of these long-term genetic contributions.

An individual's expected genetic contributions (IGC)—the proportional contribution of an individual to the gene pool at a specific point in time—is expected to stabilize over generations and enable the estimation of the genetic contributions over far longer periods [7,9,10]. Assuming, among others, random mating, non-overlapping generations, negligible inbreeding and stable population size, stabilization is predicted to occur after approximately 10 generations in a population of 1000 individuals, but longer in larger populations or if any of these assumptions are violated [7,9,11]. Largely in line with these theoretical predictions, three studies of wild vertebrate populations found IGC to be relatively stable after around eight generations [7,10,12] (though other studies have measured expected genetic contributions over shorter periods; e.g. [13]). They also found that LRS may predict variation in IGC, but the amount of variation explained varied greatly among studies (less than 1–48%; electronic supplementary material, table S1) [7,12,14]. The latter is expected as an individual's realized genetic contribution is the ultimate outcome of many factors (e.g. selection, migration, environmental stochasticity and genetic drift), all of which we expect to vary among study systems. For example, we expect LRS to be a poor predictor of IGC if long-term stochastic processes override an initial adaptive response to selection.

The degree to which LRS predicts IGC may also vary with aspects of a species's or population's life history. For example, given a similar number of generations, we could expect the correlation between LRS and IGC to be lower in species that are long-lived and reproduce over longer time periods, as due to the longer time span there is a greater likelihood that they are exposed to either stochastic mortality events (e.g. a disease outbreak) or changes in selection pressures (e.g. the appearance of a new predator, as in a study by Alif *et al.* [12]). However, thus far only species with relatively short generation times (e.g. approx. 2–4 years [7,10,12,14]) have been examined. This is at least partly for practical reasons: estimating IGC and demonstrating their stabilization in longer lived species requires individual-based data across longer time periods, which are generally more difficult to obtain.

Human genealogical data, which typically spans centuries rather than decades, provide a powerful opportunity to examine the extent to which LRS predicts long-term genetic contributions in a long-lived species with relatively long generation times. Furthermore, by comparing the predictive power of LRS to other fitness proxies, such as lifespan and the number of grandchildren, we can identify key determinants of variation in IGC. For example, annual survival is considered to be a particularly important driver of within-generation changes to the gene pool (e.g. [15]) in humans. Furthermore, lifespan is associated with increased reproductive success [16]. Hence we would expect lifespan to predict IGC, albeit probably with less accuracy than LRS as it does not directly measure reproductive output. The predictive power of LRS is also likely to vary depending on if it is conditioned on offspring survival until a certain age: in pre-demographic transition humans, infant mortality was high [17,18]. Therefore, measuring LRS as the number of offspring surviving to adulthood and not only the number born should better predict IGC. Finally, variation in both the survival and reproduction of an individual and their offspring are ultimately captured by an individual's number of grandoffspring [19], which is expected to provide a more precise predictor of IGC than LRS. Quantifying the differences in the predictive power of lifespan, number of (surviving) offspring and the number of grandoffspring will give insight into the relative importance of parental and offspring survival and reproduction in shaping IGC in humans.

The number of grandoffspring is not only expected to explain more variation in IGC (i.e. to be a more precise predictor), but it may also be less biased than LRS (i.e. more accurate). For example, LRS may overestimate IGC if there is an offspring quality versus quantity trade-off or sibling competition, causing offspring from larger families to have lower fitness [20]. Conversely, sibling cooperation (e.g. [21]) could cause LRS to underestimate IGC if individuals with many siblings have improved fitness. A first step towards identifying the underlying causes of any bias is testing if LRS systematically over- or underestimates IGC. We can do this by quantifying the relationship between an individual's LRS and the average IGC of their offspring (i.e. of siblings). If this relationship is negative, LRS overestimates the IGC of individuals with high LRS (e.g. due to quality–quantity trade-off or sibling competition), whereas a positive relationship is suggestive of LRS underestimating IGC. This may be the result of e.g. sibling cooperation [21], additive genetic variance in LRS [22] or other parental quality effects (e.g. mediated by socio-economic status) that positively affect both parental reproduction and offspring survival/reproduction.

Here, we quantify the degree to which LRS shapes pedigree-derived estimates of stabilized IGC measured after at least 8, and on average 10, (potential) generations [10] using data from a genealogical archive containing the life histories of humans from two parishes in the canton of Glarus, Switzerland. This dataset spans up to 16 generations, containing individuals born in the sixteenth to the twentieth century. We estimate IGC and infer the number of generations required to reach stabilization. We then use generalized linear mixed models (GLMMs) to examine the degree to which IGC are predicted by four fitness proxies: (i) lifespan, (ii) LRS counting all born offspring (LRS), (iii) LRS counting only offspring surviving to adulthood (LRS_SA_) and (iv) the number of grandoffspring. We then compare the predictive power of these four proxies to elucidate the importance of parental and offspring survival and reproduction in shaping IGC, and compare these results to those of previously studied bird species. Finally, we test if LRS provides a biased prediction of IGC by estimating the relationship between an individual's LRS and the average IGC of their offspring.

## 2. Methods

### (a) Dataset

We use life-history information, including an individual's year of birth, marriage and death, and the identity of its children, for individuals born or married in two parishes in the canton of Glarus, Switzerland: Linthal (46°55′ N, 9° E) and Elm (46°55′ N, 9°10′ E). The genealogical archive from which these data were extracted is predominantly based on church records but includes records for unmarried adults, children dying before reaching adulthood, and illegitimate children [23] (although these are rare, in line with expectations of historical European populations [24,25]).

The data span over four centuries, containing individuals born from 1562 to 1996. The pedigree reconstructed from these records contained 44 967 individuals, 35 882 maternities, 35 973 paternities and 89 904 full-sibling relationships. The mean maternal and paternal sibship sizes were 4.01 and 4.42, respectively. There were 8667 founders (individuals with unknown parents), and the mean and maximum pedigree depth were 6.9 and 16 generations, respectively.

During the eighteenth to twentieth centuries, population sizes of Linthal and Elm were in the ranges 994–2645 and 516–1051, respectively [26,27]. The household and family structures are representative of Central Europe as a whole (nuclear and patriarchal), with new households being formed after couples had accumulated enough wealth to get married [28]. As such, the median age-at-first reproduction for females was 25, and for 95% of individuals occurred after 19 years of age. For individuals who reproduced, the median number of offspring born was 4 (range = 1–24). Families were largely sustained through the farming of sheep and cattle, with additional earning through weaving and spinning becoming possible in the eighteenth century [29], particularly in Linthal. Over the course of the entire study period and across all individuals, the median lifespan was 49 years and 74% of individuals lived beyond age 5.

### (b) Estimation of individual genetic contributions

We estimated IGC following Hunter *et al.* [10], which uses pedigree information to estimate *expected* genetic contributions to future generations, under the *expectation* of random Mendelian segregation of alleles (e.g. each parent contributes 50% of an offspring's alleles). Hence, IGC provides an estimate of the allele copies given to descendants, and the realized contribution will vary around this expectation. The relatedness matrix, containing the relatedness coefficients between all pairs of individuals (e.g. for a parent and offspring, the relatedness coefficient is 0.5), was created in R 4.1.1 [30] using the package *nadiv* 2.17.1 [31]. These relatedness coefficients become expected genetic contributions when directionality is considered: an individual *gives* its offspring 50% of their alleles, and therefore the absolute expected genetic contribution an individual makes to its offspring is 0.5. We will henceforth refer to the individual making the expected genetic contributions as the *focal* individual and to the individual receiving the genetic contribution as the *descendant*.

IGC are equal to the expected genetic contributions proportional to the total gene pool for a given population at a given time point (i.e. all individuals alive and located in the study population). We used birth and marriage locations along with birth and death years to determine if individuals were present in the population (Linthal and Elm were analysed separately) for all individuals with a known birth year (Linthal, *n* = 19 558, 98%; Elm, *n* = 16 484, 97%; electronic supplementary material S1). To estimate IGC, we subset for each individual in each year the relationship matrix to include only the focal individual (row) and all individuals present in the specific population at that point (columns), starting at the focal individual's birth year (or arrival year if an immigrant; see electronic supplementary material S1). The total expected genetic contribution of a focal individual to the gene pool in a given year is the sum of this subset of relatedness coefficients. This was done for all the years following an individual's year of birth until 1990. Following previous studies [7,10,12,14], we did not consider IGC through non-direct descent (e.g. kin genetic contributions) by temporarily removing parental IDs of the focal individuals from the pedigree before creating the relatedness matrix. Genetic contributions were converted into IGC by dividing them by the total number of individuals present in the population in that year.

### (c) Stabilization of individual genetic contributions

Although IGC fluctuate over time, they are expected to stabilize and become representative of longer term genetic contributions [9,11,32]. Following previous work [7,12], we evaluated stabilization of IGC by grouping individuals into 10-year birth cohorts and quantifying the Pearson correlation coefficient between IGC to each subsequent year and the final year considered (1990). Ten-year cohorts were used to ensure each cohort had at least two focal individuals, the smallest sample size that allows for the calculation of a correlation coefficient. When the correlation remained above a 0.95 threshold for a period of two generations, IGC were considered to have stabilized. We defined a generation as the mean (± s.e.) parental age at offspring birth, which were 32.2 ± 0.04 and 32.1 ± 0.05 years for Linthal and Elm, respectively.

**Figure 1.**
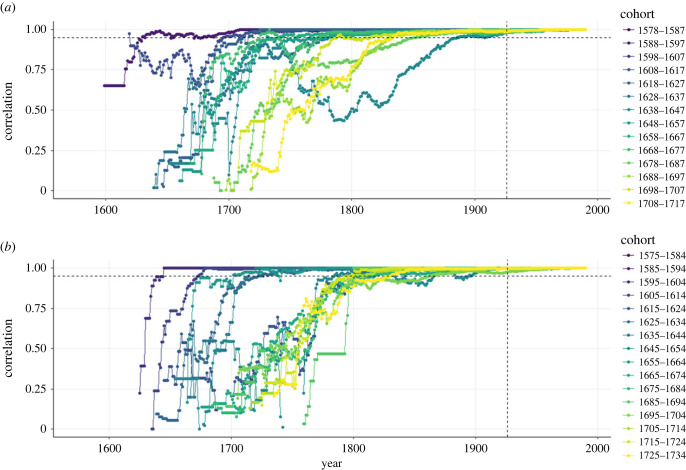
Stabilized IGC. Pearson correlation coefficients between the genetic contribution of individuals grouped into 10-year birth cohort in each year and their final year IGC. Stabilization is defined as the correlations exceeding 0.95 (horizontal dotted line) for at least two generations pre-1990 (vertical dotted line) [4]. Plots are shown for the parishes of (*a*) Linthal and (*b*) Elm. Only stabilized cohorts are shown here (born before 1718 for Linthal and 1735 for Elm), but see electronic supplementary material, figure S2.

According to this criterion, IGC had stabilized in 1990 for individuals born before 1718 in Linthal (or after 8.5 generations) and before 1734 in Elm (after 8 generations; figure 1; see electronic supplementary material, figure S2 for a comparison to non-stabilized IGC). Hence, IGC to the year 1990 from 3475 focal individuals (1605 from Linthal and 1870 from Elm) were used for further analyses. The length over which IGC were estimated was at least 274 and 257 years, and on average 10.1 and 9.9 generations (324.81(±0.86) and 319.26 (±0.93) years, for Linthal and Elm, respectively), with the birth years of focal individuals ranging between 1575 and 1734 (electronic supplementary material, figure S3).

### (d) Migration

Despite having fulfilled our criterion for stabilization, IGC will continue to change in populations with a non-zero migration rate (electronic supplementary material, figure S4). This is because immigration decreases IGC by adding to the gene pool but not to the IGC of focal individuals, thereby diluting their contribution to the gene pool. Emigration also decreases IGC and can lead to lineage extinction if emigrating offspring do not contribute to the *local* gene pool. In addition, migration will introduce variation in IGC not captured by any fitness proxies, and hence weakening their correlation with IGC.

To quantify the potential effect of migration on IGC, we classified individuals born and married in the population as residents, individuals born outside but married in the population as immigrants, and individuals born in the population but married outside as emigrants. In Linthal and Elm the vast majority of individuals were residents (62.9% and 61.5%, respectively), but both populations had a substantial proportion of immigrants (16.5% and 15.8%, respectively) and emigrants (20.6% and 22.7%). There was also a very small percentage of individuals who moved between the two parishes (from Linthal to Elm, 0.17%, and Elm to Linthal, 0.22%; see electronic supplementary material, figure S1).

To quantify how often lineage extinction was the result of descendants dispersing versus dying before reproduction, we calculated for each focal individual the percentages of now deceased descendants (traced using the *visPedigree* [33] package) that successfully continued the lineage (i.e. reproduced in the population), did not reproduce in the population, and dispersed (emigrated) out of the population.

### (e) Fitness proxies

We considered the following fitness proxies: lifespan (the difference between the death date and birth date), LRS (lifetime number of offspring produced), LRS_SA_ (lifetime number of offspring surviving to adulthood) and the number of grandoffspring (total number of offspring of an individual's offspring). Adulthood was defined as the sex-specific fifth percentile of age-at-first reproduction for the whole dataset (females: 19.1 years, males: 21.2 years). We estimated lifespan, LRS and LRS_SA_ for all individuals for which we had an estimated IGC and with known birth and death dates (*n* = 2358), including individuals that died before adulthood. For the number of grandoffspring, we additionally required that the individual's offspring also had their complete life-history recorded (*n* = 2358).

### (f) Statistical analyses

We used GLMMs to examine the relationship between IGC and the four fitness proxies. We used a zero-inflated beta model in which the zero-inflated part of the model modelled the probability of an individual's IGC to the present-day gene pool being equal to zero (i.e. the probability of lineage extinction) using a logit-link function. It should be noted that this models the probability of an individual having no living descendants in the focal population, not the extinction probability of a specific gene. The distribution of the non-zero proportional genetic contributions was modelled using a beta distribution.

We controlled for differences in mean IGC, for example due to differences in population size, between both parishes (Linthal or Elm) and the sexes (female or male) by including these as categorical fixed effects. An individual's 10-year parish-specific birth cohort was fitted as a random intercept to control for temporal variation in mean IGC. We furthermore included a random slope for the effect of each of the fitness proxies to allow their relationship with IGC to vary among parish-specific birth cohorts. Initially a two-way interaction between sex and parish was included, but this was removed if non-significant to aid the interpretation of first-order effects. Model structures were the same for the zero-inflated and beta parts of the model. Counting only individuals that were informative for all predictors, the sample size for these models was 2230.

To quantify how much variation in IGC each fitness proxy explained, we estimated the Bayesian R-squared for each of our models [34]. The significance of the differences in Bayesian R-squared values was evaluated through finding the mode and 95% credible intervals of the difference between the R-squared values of the models being compared (Δ*R*^2^) and seeing if these 95% credible intervals overlapped 0.

We quantify the bias in LRS in predicting IGC by examining the slope of the relationship between the LRS of an individual and the mean IGC of their offspring. Here we used the same individuals as before, but excluding non-reproducing individuals, leaving 1256 individuals. For this model, we performed a beta regression (with no zero-inflated distribution included) controlling for the same confounding fixed and random effects structures as above. Beta regressions require response variables to non-zero values and we therefore added 10^−10^ to all mean offspring genetic contributions. Here, no relationship would indicate LRS is an unbiased predictor of IGC. We additionally examined if the lifespan of parents was an important covariate, as offspring whose parents died younger might receive less parental care, potentially impacting IGC of their offspring.

Both zero-inflated beta and beta models were implemented in the R package *brms* (2.16.1 [35]) using the Markov chain Monte Carlo sampler Rstan (2.21.2 [36]) using R (4.0.2 [30]). For each model, we ran four runs of 6000 iterations across four cores, sampling every 10 iterations, after a warm-up of 2000 iterations. We set the delta parameter to 0.95 to aid convergence. Default priors were used: flat for all fixed effects and a student's t distribution for random effects. Convergence of models was confirmed based on R hat parameters and Monte Carlo standard errors being approximately 1 and 0, respectively. The *pp_check* function was used to check that simulated data from the model matched the original data well. We used the probability of Direction (*pd*) [37] (the percentage of the posterior distribution that has the same sign as the median) to infer statistical significance. In line with Makowski *et al.* [37], we classified *pd* values as follows: 0.95–0.975 = trend effect; 0.975–0.99 = significant; greater than 0.99 = highly significant. For random effects, *pd* is not applicable and no significance criteria were used. Figures were created using the packages *brms, ggplot2* (3.3.5, [38]) and *ggpubr* (0.4.0 [39]).

## 3. Results

### (a) Individual genetic contributions

We estimated the IGC for 3475 individuals (1605 from Linthal and 1870 from Elm), born between 1575 and1735, to the individuals making up the gene pool of the parishes of Linthal and Elm in 1990. The probability of an individual's lineage going extinct was high, with 73% of individuals having an IGC of zero to the 1990 population (electronic supplementary material, figure S5*a*). The majority of extinctions are because an individual did not survive to reproductive age (23.4%), survived until reproductive age but had no offspring (43.5%), had offspring but none survived to adulthood (45.3%) or had surviving offspring but no grandoffspring (52.7%) (electronic supplementary material, figure S5). This leaves approximately 20% of the individual lineage extinctions occurring after individuals had at least one grandchild. Over all individuals included in the analysis, a median of 15.9% of their descendants reproduced and thereby continued the lineage, and 14.6% of the descendants had not yet reproduced but were still alive. This leaves 69.7% of the descendants who failed to continue the lineage, and of these, a median of 40.3%, due to emigration rather than death without reproducing. Individuals whose lineages did not go extinct on average contributed 0.1% of the genetic material present in the population in 1990 (electronic supplementary material, figure S5*a*), although one male contributed 0.6% of the Linthal gene pool.

**Figure 2.**
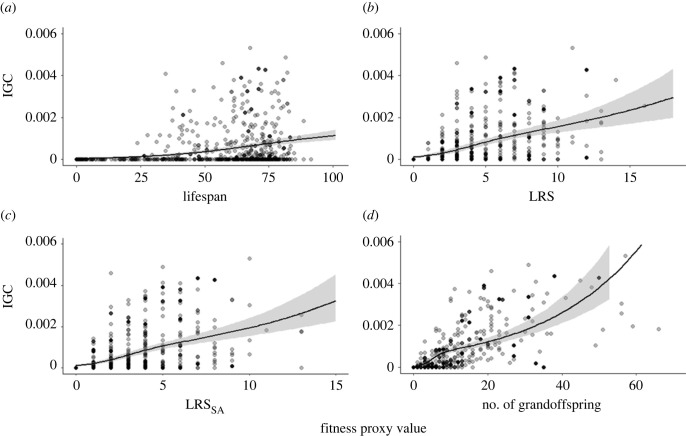
The relationship between IGC and four fitness proxies: (*a*) lifespan, (*b*) LRS, (*c*) LRS_SA_ and (*d*) the number of grandoffspring. The plots were produced using the *conditional_effects()* function from the R package *brms* to standardize points across values for covariates. Shaded areas indicate 95% credible intervals of the model estimate. Data are conditioned on the mean values of the other predictors (birth cohort, parish and sex). Data points are partially transparent to aid visualization.

Lifespan, LRS, LRS_SA_ and the number of grandoffspring were positively associated with IGC (Beta distribution, *pd* > 0.975; table 1; figure 2). We also found a negative effect of any of the fitness proxies on the probability of an individuals' lineage going extinct (zero-inflated distribution, *pd* > 0.975; table 1).

**Table 1.** Output from four zero-inflated beta models of IGC with different fitness proxies included: lifespan, LRS, LRS_SA_ and the number of grandoffspring. Fixed and random effect estimates (posterior distribution median [95% credible intervals] are provided for both the zero-inflated and beta distributions. Significant effects (probability of direction > 0.975) are in italics. Non-significant two-way interactions were removed from the models. Model results with non-significant interactions included are shown in the electronic supplementary material, table S2.

	lifespan		LRS		LRS_SA_		number of grandoffspring	
	zero inflated	beta	zero inflated	beta	zero inflated	beta	zero inflated	beta
fixed effects								
intercept	*3.248 [2.882–3.628]*	*−7.171 [−7.468–−6.880]*	*2.145 [1.880–2.427]*	*−7.177 [−7.328–−7.022]*	*2.219 [1.926–2.521]*	*−7.264 [−7.418–−7.118]*	*3.032 [2.623–3.415]*	*−7.442 [−7.564–−7.322]*
[fitness proxy]	*−0.054 [−0.059–−0.048]*	*0.006 [0.002–0.010]*	*−0.587 [−0.659–−0.515]*	*0.065 [0.047–0.082]*	*−0.796 [−0.922–−0.684]*	*0.098 [0.075–0.119]*	*−0.591 [−0.711–−0.475]*	*0.035 [0.031–0.039]*
birth parish (Linthal)	−0.037 [−0.351–0.297]	*−0.312 [−0.448–−0.162]*	0.319 [−0.053–0.718]	*−0.403 [−0.535–−0.263]*	0.255 [−0.101–0.631]	**−***0.396 [−0.533–−0.260]*	0.220 [−0.323–0.781]	*−0.489 [−0.63–−0.349]*
sex (male)	−0.154 [−0.371–0.049]	0.079 [−0.029–0.188]	0.138 [−0.111–0.389]	0.050 [−0.057–0.157]	0.115 [−0.125–0.362]	0.063 [−0.045–0.169]	0.141 [−0.241–0.493]	0.057 [−0.047–0.158]
random effects								
parish-specific birth cohort (random intercept)	0.217 [0.009–0.626]	0.110 [0.004–0.371]	0.153 [0.006–0.415]	0.069 [0.002–0.212]	0.170 [0.008–0.465]	0.066 [0.002–0.187]	0.281 [0.014–0.759]	0.061 [0.003–0.165]
parish-specific birth cohort × fitness proxy (random slope)	0.006 [0.001–0.014]	0.002 [0.000–0.006]	0.138 [0.069–0.223]	0.011 [0.001–0.031]	0.268 [0.172–0.402]	0.014 [0.001–0.034]	0.269 [0.173–0.402]	0.004 [0–0.009]

IGC (distribution and extinction probability) were dependent upon several other factors. First, individuals born in Linthal had lower IGC, probably because of its larger population size (all models, beta distribution, *pd* > 0.975; table 1). In line with this, there was no difference in probability of lineage extinction (zero-inflated distribution, *pd* < 0.975; table 1). There were no interactions between these effects and sex (*pd* < 0.975; electronic supplementary material, table S2) and no differences between males and females were found (beta and zero-inflated distribution, *pd* < 0.975; table 1). Further, we found that IGC of individuals varied among birth cohorts (both in their extinction probability and in the non-zero IGC values; see random effects; table 1). There was also variation among birth cohorts in the slope of the relationship between each fitness proxy and IGC, but except for the slope of the relationship between probability of lineage extinction and LRS, LRS_SA_ and the number of grandoffspring, this variation was small. Finally, a supplementary analysis showed that the proportion of offspring migrating was associated with lower IGC and higher extinction probabilities, but accounting for this did not substantially change the predictive power of the models (electronic supplementary material S2 and table S3).

### (b) How well do fitness proxies predict individual genetic contributions?

Although all fitness proxies predicted IGC, we found that they significantly varied in their predictive power. As expected, the number of grandoffspring explained most variation in IGC (*R*^2^ = 57.3%; table 2), explaining 44.3 percentage points more variation than lifespan, 29.8 percentage points more than LRS and 25.2 percentage points more than LRS_SA_ (table 2). Contrary to expectations, the difference in predictive power between LRS and LRS_SA_ was very small (Δ*R*^2^ = 2.7%, Δ95% Credible Intervals (CrI)=−1.8% – 9.2%; table 2). A null model containing no fitness proxy but all other first-order fixed and effects and random effects explained only 1.4% (95% CrI = 0.9%–2.2%) of the variation in IGC.

**Table 2.** On the diagonal, Bayesian *R*^2^ values (*R*^2^ and 95% credible intervals) for models containing either lifespan, LRS, LRS_SA_ or the number of grandoffspring and any other significant covariates retained in the model. Pairwise Pearson correlation coefficients between fitness proxies are shown above the diagonal (also see electronic supplementary material, figure S6) and the difference in Bayesian *R*^2^ values are shown below the diagonal (Δ*R*^2^ and Δ95% credible intervals). Δ95% credible intervals that do not overlap with zero are in italics.

	lifespan	LRS	LRS_SA_	the number of grandoffspring
lifespan	13.2% [10.8–15.8]	0.55	0.54	0.43
LRS	*14.8% [10.5–19.1*	27.9% [24.2–31.6]	0.94	0.71
LRS_SA_	*19.2% [14.5–23.7]*	2.7% [−1.8–9.2]	32% [27.8–36.0]	0.74
number of grandoffspring	*44.3% [39.5–48.2]*	*29.8% [24.2–34.8]*	*25.2% [19.9–31.0]*	57.3% [53.5–60.8]

### (c) Is lifetime reproductive success an unbiased estimate of individual genetic contributions?

**Figure 3.**
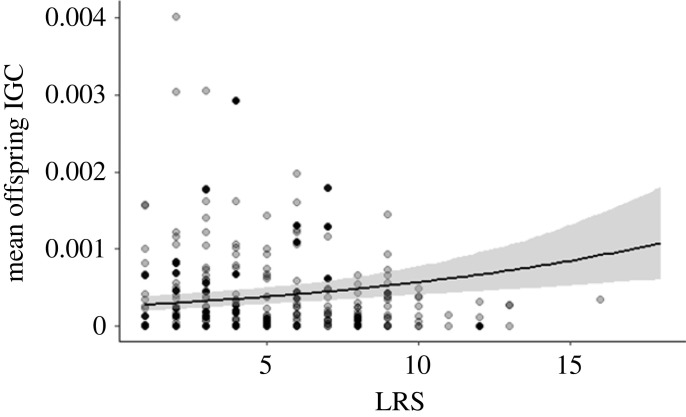
The relationship between mean offspring IGC and LRS. The plots were produced using the *conditional_effects()* function from the R package *brms* to standardize points across values for covariates. Shaded areas indicate 95% credible intervals. Data points are partially transparent to aid visualization.

The *per capita* IGC of an individual's offspring increased with LRS, but the slope of this relationship was very shallow (*pd* > 0.975, posterior mode = 0.070, 95% CrI = 0.052–0.089; figure 3). This finding suggests that LRS slightly underestimates IGC in larger family sizes. Furthermore, individuals who lived longer had offspring with higher IGC (*pd* > 0.975, posterior mode = 0.010, 95% CrI = 0.006–0.014). As before, and likely due to population size differences, mean IGC of offspring was lower for individuals born in Linthal (*pd* > 0.975, posterior mode = −0.162, 95% CrI = −0.339–0.011) but sex differences showed only trend effects and the offspring of males did not have lower mean IGC (*pd* = 0.962, posterior mode = −0.066, 95% CrI = −0.173–0.04). No interactions were significant (*pd* < 0.975; electronic supplementary material, table S4).

Finally, we found that variance in the mean IGC of offspring was explained by their parents' birth cohort (posterior mode = 0.163, 95% CrI = 0.032–0.306). The parent's birth cohort also affected the slope of relationship between LRS and mean offspring IGC but this variation was relatively small (posterior mode = 0.014, 95% CrI = 0.001–0.038).

## 4. Discussion

We quantified the extent to which LRS and other fitness proxies predict stabilized IGC measured after approximately 10 generations (321 years), in historical humans from the Swiss Canton of Glarus. We found that LRS predicted 28% of the variation in IGC, showing that reproductive success shapes the long-term genetic contributions of individuals even in a population of a long-lived species with appreciable migration that has experienced large and rapid changes in its environment.

We have shown that different fitness proxies varied in their predictive power of IGC (table 2), allowing us to identify the components of an individual's life history that are most important in determining its IGC. Overall, the model containing the number of grandoffspring explained 57% of variation in IGC, whereas the next best fitness proxy (LRS_SA_) explained only 32%, followed by LRS (28%) and lifespan (13%). This is broadly in line with results based on genetic contributions estimated over four generations in nineteenth-century Sweden [40]. The number of grandoffspring explaining most variation was expected, as the number of grandoffspring incorporates the most information about the life history of an individual. However, together with our finding that LRS_SA_ and LRS explain a similar amount of variation in IGC (28% versus 32%) and that lifespan explains only 13% of the variation in IGC, this suggests that offspring mating and reproduction is a much greater determinant of IGC than survival (of both offspring and the individual themselves), even in a population with substantial childhood mortality (electronic supplementary material, figure S1). The lack of a significant increase in the predictive power of LRS when accounting for infant mortality could be due to the replacement effect that can occur in humans where the death of a child during childhood results in another child being born soon after and the number of children surviving to adulthood to be largely unaffected. Finally, although we found no difference between the sexes, the highest IGC were for multiply-marrying males whose first wives died around the age of menopause, allowing the widowers to remarry a younger female and achieve a LRS (and IGC) greater than males who did not remarry.

Although the number of grandoffspring will naturally be a more precise predictor of IGC than LRS because it is closer in time to our estimates of IGC, and therefore incorporates more of the stochasticity that influences IGC, it is not necessarily the most useful fitness proxy. First, although statistically significant, we found only a weak relationship between an individual's LRS and the average IGC of its offspring, showing that LRS is a relatively unbiased measure of IGC. The fact that the association is positive suggests that the increase in predictive power between LRS and number of grandoffspring is not due to LRS being a biased predictor. Albeit small, the positive relationship between LRS and *per capita* IGC argues against the existence of an offspring quality–quantity trade-off, which has been previously found in humans [41–43]. Instead it is somewhat suggestive of positive sibling effects, perhaps due to alloparenting [21], or an overriding effect of parental quality and resources (e.g. through socio-economic status) (e.g. [44,45]) or additive genetic variance in LRS [22]. Second, there are practical reasons that limit the utility of the number of grandoffspring as a fitness proxy: Not only is it more sampling intensive, reliably counting the number of grandoffspring may not be feasible if a significant proportion of the population disperses outside of the study site, or offspring cannot be linked to parents once they have reached independence. Third, the number of grandoffspring confounds the fitness of multiple individuals, which can be problematic when estimating the strength of phenotypic selection [3–5]. All things considered, our results therefore strengthen the case for LRS as an evolutionary relevant and relatively unbiased fitness proxy when it comes to the study of selection in humans, assuming our findings are representative for other populations and time periods.

Although at first sight high, our finding that 70% of individual lineages went extinct over the study period is similar to that found in previous studies on pedigreed populations of birds, which reported extinction probabilities of 61–71% (electronic supplementary material, table S1), and comparable with levels of lineage extinction in bighorn sheep [13], and in humans after four generations in Sweden [46]. The main difference between our results and those for the three bird studies [7,12,14] was that when we measured LRS at a later point in the offspring's life (i.e. LRS_SA_), the predictive power of LRS for IGC did not increase greatly. This is in contrast with, for example [14], which found that offspring survival was a key determinant of reproductive success. Our results therefore suggest that despite substantial infant mortality, offspring survival to adulthood is a less important determinant of IGC than mating and reproductive success in humans.

The amount of variation in IGC explained by LRS measured close to birth (28%) in this study was similar to previous findings for song sparrows and scrub-jays (37% and 32%, respectively), but higher than for house sparrows (0–4%). While the role of migration in the latter island population is likely to be small, the low explanatory power of LRS is potentially due to a population bottleneck that occurred between the timepoint when fitness proxies were measured and when IGC was estimated [12]. This could have caused stochastic mortality resulting in low predictive power of fitness proxies. Another factor explaining different findings across populations is the role of stochasticity in driving variation in LRS itself. LRS is influenced by both environmental and genetic components with the environment contributing most of the variation [47], including in humans [48]. In species where the environment determines less variation in LRS, LRS would be expected to be a greater predictor of IGC [7,8]. Here, we showed that environmental effects were an important factor, with non-negligible variation in IGC being explained by an individual's birth cohort (table 1). Further, as mean LRS values decrease, there is a greater likelihood of lineages going extinct due to stochasticity, drift or dispersal [7,49], which perhaps partially explains the relatively high rates of lineage extinction in this study (70% versus 61–71%; electronic supplementary material, table S1). Although these species also differ in numerous other ways. However, future studies could examine if this phenomenon is detectable across the human fertility transition towards lower LRS. In summary, there are both similarities and differences across study systems, but the small number of species and the lack of different human populations (across cultures) studied limits broader extrapolation.

The majority of the variation in IGC (72%) remained unexplained in the model containing LRS, with migration being a contributing factor to this unexplained variation: First, there were significant levels of both immigration and emigration (electronic supplementary material, figure S1), and both are expected to decouple LRS and IGC. Indeed, the dispersal of descendants of ancestral individuals is a particularly important driver of local lineage extinction. Although migration is also expected to reduce the stabilization times relative to theoretical expectations, we observed stabilization times lower than theory predicts [9]. One explanation for this is that the effective population size (number of breeding individuals) is far lower than the total population size, for example because a significant proportion of individuals did not reproduce (see electronic supplementary material, figures S1 and S5). However, other explanations (e.g. non-random mating according to social class, drift and fluctuating selection) are also possible, and it is clear that we need to further our understanding of the determinants of the time until stabilization of IGC in natural populations. Enumerating the relative contributions of these factors across different systems (or using simulations, e.g. [50]) should be a target of future work.

Although still in its infancy, the use of pedigree data to estimate long-term genetic contributions opens a range of exciting avenues. Building on our work using human genealogical data, and the work on non-human animals by others [7,12,14], future work would benefit from further exploration of the similarities and differences among the different methodologies at our disposal, and between gene-dropping methods [7,12,14] and expected genetic contributions (e.g. [10]; this study) in particular, as the two do not necessarily equate. Furthermore, while our study has highlighted the ability of human genealogical data to provide insight into human evolution [51,52], and the estimation of fitness more broadly, applying these methods to similar data for an array of human populations (see [53] for a review) will allow us to quantify the degree to which these findings hold across cultures, environments and time.

## Data Availability

All data and R scripts necessary for replicating the analysis can be accessed at Dataverse https://doi.org/10.34894/P2ETYZ [54]. The data are provided in the electronic supplementary material [55].

## References

[1] Fisher RA. 1930 The genetical theory of natural selection. Oxford, UK: Oxford University Press.

[2] Clutton-Brock TH. 1988 Reproductive success: studies of individual variation in contrasting breeding systems. Chicago, IL: University of Chicago Press.

[3] Wolf JB, Wade MJ. 2001 On the assignment of fitness to parents and offspring: whose fitness is it and when does it matter? J. Evol. Biol. **14**, 347-356.

[4] Hadfield JD, Thomson CE. 2017 Interpreting selection when individuals interact. Methods Ecol. Evol. **8**, 688-699. (10.1111/2041-210X.12802)

[5] Hadfield J. 2012 The quantitative genetic theory of parental effects. In The evolution of parental care (eds, NJ Royle, PT Smiseth, M Kölliker), pp. 267-284. Oxford, UK: Oxford University Press.

[6] Kingsolver JG, Hoekstra HE, Hoekstra JM, Berrigan D, Vignieri SN, Hill CE, Hoang A, Gibert P, Beerli P. 2001 The strength of phenotypic selection in natural populations. Am. Nat. **157**, 245-261. 18707288 10.1086/319193

[7] Reid JM, Nietlisbach P, Wolak ME, Keller LF, Arcese P. 2019 Individuals’ expected genetic contributions to future generations, reproductive value, and short-term metrics of fitness in free-living song sparrows (*Melospiza melodia*). Evol. Lett. **3**, 271-285. (10.1002/evl3.118) 31171983 PMC6546383

[8] Brommer JE, Gustafsson L, Pietiäinen H, Merilä J. 2004 Single-generation estimates of individual fitness as proxies for long-term genetic contribution. Am. Nat. **163**, 505-517. 15122499 10.1086/382547

[9] Barton NH, Etheridge AM. 2011 The relation between reproductive value and genetic contribution. Genetics **188**, 953-973. (10.1534/genetics.111.127555) 21624999 PMC3176105

[10] Hunter DC, Pemberton JM, Pilkington JG, Morrissey MB. 2019 Pedigree-based estimation of reproductive value. J. Hered. **110**, 433-444. (10.1093/jhered/esz033) 31259373

[11] Chang JT. 1999 Recent common ancestors of all present-day individuals. Adv. Appl. Probab. **31**, 1002-1026. (10.1017/S0001867800009587)

[12] Alif Ž, Dunning J, Chik HYJ, Burke T, Schroeder J. 2022 What is the best fitness measure in wild populations? A case study on the power of short-term fitness proxies to predict reproductive value. PLoS ONE **17**, e0260905. 35452482 10.1371/journal.pone.0260905 PMC9032343

[13] Van de Walle J, Larue B, Pigeon G, Pelletier F. 2022 Different proxies, different stories? Imperfect correlations and different determinants of fitness in bighorn sheep. Ecol. Evol. **12**, 1-12. 10.1002/ece3.9582 PMC9731912 36514553

[14] Chen N, Juric I, Cosgrove EJ, Bowman R, Fitzpatrick JW, Schoech SJ, Clark AG, Coop G. 2019 Allele frequency dynamics in a pedigreed natural population. Proc. Natl Acad. Sci. USA **116**, 2158-2164. (10.1073/pnas.1813852116) 30598449 PMC6369762

[15] Scranton K, Lummaa V, Stearns SC. 2016 The importance of the timescale of the fitness metric for estimates of selection on phenotypic traits during a period of demographic change. Ecol. Lett. **19**, 854-861. 27230740 10.1111/ele.12619

[16] Lahdenperä M, Lummaa V, Helle S, Tremblay M, Russell AF. 2004 Fitness benefits of prolonged post-reproductive lifespan in women. Nature **428**, 178-181. (10.1038/nature02367) 15014499

[17] Corbett S, Courtiol A, Lummaa V, Moorad J, Stearns S. 2018 The transition to modernity and chronic disease: mismatch and natural selection. Nat. Rev. Genet. **19**, 419-430. (10.1038/s41576-018-0012-3) 29743650

[18] Wells JCK, Stock JT. 2007 The biology of the colonizing ape. Yearb Phys. Anthropol. **50**, 191-222. 10.1002/ajpa.20735 18046751

[19] Hunt J, Bussière LF, Jennions MD, Brooks R. 2004 What is genetic quality? Trends Ecol. Evol. **19**, 329-333. 16701279 10.1016/j.tree.2004.03.035

[20] Lack D. 1947 The significance of clutch-size. Ibis (Lond 1859) **89**, 302-352. (10.1111/j.1474-919X.1947.tb04155.x)

[21] Nitsch A, Faurie C, Lummaa V. 2013 Are elder siblings helpers or competitors? Antagonistic fitness effects of sibling interactions in humans. Proc. R. Soc. B **280**, 20122313. (10.1098/rspb.2012.2313) PMC3574445 23173210

[22] Bonnet T et al. 2022 Genetic variance in fitness indicates rapid on-going adaptive evolution in wild animals. Science **376**, 1012-1016. 35617403 10.1126/science.abk0853

[23] Kubly-Müller J. 1912 Die Genealogien-Werke des Kantons Glarus. Archiv für Heraldik **4**, 164-187.

[24] Larmuseau MHD, Matthijs K, Wenseleers T. 2016 Cuckolded fathers rare in human populations. Trends Ecol. Evol. **31**, 327-329. (10.1016/j.tree.2016.03.004) 27107336

[25] Larmuseau MHD et al. 2019 A historical-genetic reconstruction of human extra-pair paternity. Curr. Biol. **29**, 4102-4107.e7. (10.1016/j.cub.2019.09.075) 31735678

[26] Marti-Weissenbach K. 2020 Linthal Historisches Lexikon der Schweiz (HLS). See https://hls-dhs-dss.ch/de/articles/000770/2020-11-19/

[27] Marti-Weissenbach K. 2020 Elm. Historisches Lexikon der Schweiz (HLS). See https://hls-dhs-dss.ch/fr/articles/000762/2020-11-19/

[28] Wall R. 1983 Introduction. In Family forms in historic Europe (eds P Laslett, R Wall), pp. 1-63. Cambridge, UK: Cambridge University Press.

[29] Laupper H, Peter Schindler M, Tremp E, Kamm R, Marti-Weissenbach K, Head-König AL et al. 2017 Glarus (Kanton). Historisches Lexikon der Schweiz (HLS). See https://hls-dhs-dss.ch/de/articles/007374/2017-05-30/

[30] R Core Team. 2020 R: a language environment for statistical computing. Vienna, Austria: R Foundation for Statistical Computing. See http://www.r-project.org/.

[31] Wolak ME. 2012 nadiv: an R package to create relatedness matrices for estimating non-additive genetic variances in animal models. Methods Ecol. Evol. **3**, 792-796. (10.1111/j.2041-210X.2012.00213.x)

[32] Grafen A. 2006 A theory of Fisher's reproductive value. J. Math. Biol. **53**, 15-60. (10.1007/s00285-006-0376-4) 16791649

[33] Luan S. 2022 Vispedigree: tidying and visualization for animal pedigree. See https://github.com/luansheng/visPedigree

[34] Gelman A, Goodrich B, Gabry J, Vehtari A. 2019 R-squared for Bayesian regression models. Am. Stat. **73**, 307-309. (10.1080/00031305.2018.1549100)

[35] Bürkner PC. 2018 Advanced Bayesian multilevel modeling with the R package brms. R J. **10**, 395-411. (10.32614/RJ-2018-017)

[36] Stan Development Team. 2020 RStan: the R interface to Stan. See http://mc-stan.org/.50.

[37] Makowski D, Ben-Shachar MS, Chen SHA, Lüdecke D. 2019 Indices of effect existence and significance in the Bayesian Framework. Front. Psychol. **10**, 1-14. (10.3389/fpsyg.2019.02767) 31920819 PMC6914840

[38] Wickham H. 2016 Ggplot2: elegant graphics for data analysis. See https://ggplot2.tidyverse.org

[39] Kassambara A. 2020 Ggpubr: ‘ggplot2’ based publication ready plots. See https://cran.r-project.org/package=ggpubr

[40] Kolk M, Skirbekk V. 2022 Fading family lines- women and men without children, grandchildren and great-grandchildren in 19th, 20th and 21st century northern Sweden. Adv. Life Course Res. **53**, 100481. (10.1016/j.alcr.2022.100481) 36652207

[41] Lynch RF. 2016 Parents face quantity-quality trade-offs between reproduction and investment in offspring in Iceland. R. Soc. Open Sci. **3**, 160087. (10.1098/rsos.160087) 27293787 PMC4892449

[42] Bolund E, Lummaa V. 2017 The effects of resource availability and the demographic transition on the genetic correlation between number of children and grandchildren in humans. Heredity (Edinb) **118**, 186-192. (10.1038/hdy.2016.81) 27624115 PMC5234483

[43] Lawson DW, Alvergne A, Gibson MA. 2012 The life-history trade-off between fertility and child survival. Proc. R. Soc. B **279**, 4755-4764. (10.1098/rspb.2012.1635) PMC3497086 23034700

[44] Van Noordwijk AJ, De Jong G. 1986 Acquisition and allocation of resources: their influence on variation in life history tactics. Am. Nat. **128**, 137-142. (10.1086/284547)

[45] Reznick D, Nunney L, Tessier A. 2000 Big houses, big cars, superfleas and the costs of reproduction. Trends Ecol. Evol. **15**, 421-425. (10.1016/S0169-5347(00)01941-8) 10998520

[46] Goodman A, Koupil I, Lawson DW. 2012 Low fertility increases descendant socioeconomic position but reduces long-term fitness in a modern post-industrial society. Proc. R. Soc. B **279**, 4342-4351. (10.1098/rspb.2012.1415) PMC3479798 22933371

[47] Mousseau TA, Roff DA. 1987 Natural selection and the heritability of fitness components. Heredity (Edinb) **59**, 181-197. 3316130 10.1038/hdy.1987.113

[48] Stearns SC, Byars SG, Govindaraju DR, Ewbank D. 2010 Measuring selection in contemporary human populations. Nat. Rev. Genet. **11**, 611-622. 20680024 10.1038/nrg2831

[49] Gravel S, Steel M. 2015 The existence and abundance of ghost ancestors in biparental populations. Theor. Popul. Biol. **101**, 47-53. 25703300 10.1016/j.tpb.2015.02.002

[50] Borger MJ, Komdeur J, Richardson DS, Weissing FJ. 2022 Putting life history theory to the test: the estimation of reproductive values from field data. *bioRxiv* 2022.03.09.483591. 1–23. (10.1101/2022.03.09.483591) PMC10516676 37744170

[51] Brosnan SF, Postma E. 2017 Humans as a model for understanding biological fundamentals. Proc. R. Soc. B **284**, 20172146. (10.1098/rspb.2017.2146) PMC5745415 29237858

[52] Briga M, Griffin RM, Berger V, Pettay JE, Lummaa V. 2017 What have humans done for evolutionary biology? Contributions from genes to populations. Proc. R. Soc. B **284**, 20171164. (10.1098/rspb.2017.1164) PMC5698635 29118130

[53] Bolund E, Hayward A, Lummaa V. 2016 Life-history evolution, human. Encycl. Evol. Biol. **2** 328-334. (10.1016/B978-0-12-800049-6.00097-4)

[54] Young EA, Chesterton E, Lummaa V, Postma E, Dugdale HL. 2023 Data from: The long-lasting legacy of reproduction: lifetime reproductive success shapes expected genetic contributions of humans after ten generations. *Dataverse*. (10.34894/P2ETYZ) PMC10170207 37161329

[55] Young EA, Chesterton E, Lummaa V, Postma E, Dugdale HL. 2023 The long-lasting legacy of reproduction: lifetime reproductive success shapes expected genetic contributions of humans after ten generations. *Figshare*. (10.6084/m9.figshare.c.6619769) PMC10170207 37161329

